# Editorial: Advances in Memory and Cognitive Decline Associated with Aging and Alzheimer’s Disease

**DOI:** 10.3390/brainsci16101064

**Published:** 2026-10-01

**Authors:** Pavan Kumar

**Affiliations:** Department of Anatomy and Cell Biology, College of Medicine, The University of Illinois at Chicago, Chicago, IL 60612, USA; kumarpa@uic.edu

## 1. Introduction

Alzheimer’s disease is a progressive neurodegenerative disease in the elderly population. AD and ADRD (Alzheimer’s disease-related dementia) are a global burden. It is categorized into two types. Early-onset (familial) and late-onset (sporadic). Sporadic AD accounts for 95% of cases and typically begins after age 65. According to the Alzheimer’s Disease Association report (2025), 7.2 million Americans age 65 and older are living with AD dementia. In 2000 and 2022, deaths from AD increased by 142% compared to stroke, heart disease, and HIV [1]. Alarmingly, Asia experienced a 250.44% increase in AD cases [2]. According to the World Health Organization study, 55 million people with dementia impose an economic burden of 1.3 trillion dollars globally, and the anticipated projection will be 152.8 million in 2050 [3,4].

In our Special Issue, we focused on advances in understanding memory and cognitive decline associated with aging and AD. Published articles focus on natural compounds, artificial intelligence (AI) tools, non-invasive methods, and experimental animal models to better understand AD and ADRD.

## 2. Summary of Accepted Papers

Exarchos et al.’s (Contribution 1) study on the geographic distribution of the future projection of mild cognitive impairment (MCI) in Greece shows that regional disparities are driving an accelerating public health crisis. The number of patients with cognitive impairment increased between 1991 and 2023. Exarchos et al. (Contribution 1) used data from the Hellenic Longitudinal Investigation of Aging and Diet (HELIAD) and the Hellenic Statistical Authority (ELSTAT) census to track changes in patient counts over several decades. MCI cases increased from 177,898 in 1991 to 311,189 in 2023, and dementia cases more than doubled in the same period, from 103,535 to 206,939; this upward trajectory is expected to continue. By 2035, MCI cases are projected to reach 375,000, and dementia cases 250,000; by 2050, the numbers are expected to hit 440,000 for MCI and 310,000 for dementia. The study developed distribution heatmaps to help policymakers identify these high-burden “hotspot” regions for targeted resource allocation.

Fu et al. (Contribution 2) proposed a novel deep learning framework, the multi-model deep domain adaptation (MM-DDA) model, to improve the diagnostic accuracy of Alzheimer’s disease (AD) classification by integrating diverse imaging data and addressing inconsistencies across datasets. The primary objective of the research was to overcome the “domain shift” problem, which occurs when medical imaging data from various devices have inconsistent distributions, making traditional models less effective on new datasets. MM-DDA consists of three core components that integrate MRI and PET data: (1) Feature Encoding Module, which uses four parallel 3D convolutional neural networks to extract both detailed and abstract features from MRI and PET scan images. (2) Multi-Head Attention Feature Fusion Module, which uses a multi-head attention mechanism that dynamically adjusts weights to fuse structural and functional brain information more effectively. (3) Domain Transfer Module, which reduces the distributional discrepancies between the source and target domains by employing adversarial learning. In experimental testing using Alzheimer’s Disease Neuroimaging Initiative (ADNI) datasets, the model demonstrated high diagnostic performance. AD vs. Normal control (CN), Progressive MCI (pMCI) vs. Stable MCI (sMCI), and MCI vs. CN reached 94.73%, 81.8%, and 85.45%, respectively. The study concludes that MM-DDA consistently outperforms single-model methods and other existing domain adaptation techniques and provides new tools and methods for clinical diagnosis.

Macoir et al. (Contribution 3) explore the utility of a specialized memory test in identifying subtle cognitive deficits in individuals who report memory concerns but perform normally on standard assessments. The primary goal of this research was to investigate whether the Face-Name Associative Recognition Test (FNART) could provide objective evidence of associative memory impairment in individuals with Subjective Cognitive Decline (SCD). The study involved 69 adults with SCD and 69 healthy controls (HC). The FNART procedure required participants to encode 32 face-first name pairs, followed by an interference phase and a recognition phase in which they matched the correct name to the corresponding face. The healthy control group performed better than the SCD group in the total number of correct responses, demonstrating that FNART is sensitive enough to objectify memory difficulties that standard tests often miss. In the serial position analysis, researchers also found that the SCD group performed significantly worse than the HC group only for items in the middle of the list. While primacy and recency effects were intact, SCD patients struggled with middle items, indicating a specific weakness in associative episodic memory. The study concludes that associative memory is more vulnerable in individuals with SCD than in HC, and that FNART is an effective, simple-to-administer tool for identifying cognitive decline in the preclinical stage of AD.

A review by Tang et al. (Contribution 4) explores the critical role of the TH17/Treg axis in the etiology of AD and evaluates how natural medicines can restore this balance to mitigate neuroinflammation. An imbalance between pro-inflammatory Th17 cells and anti-inflammatory regulatory T (Treg) cells plays a central role in driving chronic neuroinflammation and cognitive decline in AD. Th17 cells exacerbate neuronal damage by releasing pro-inflammatory cytokines such as IL-17, whereas Treg cells provide neuroprotection by secreting anti-inflammatory mediators such as IL-10 and TGF-β. Disruption of the blood–brain barrier allows infiltration of these peripheral immune cells into the CNS, where they trigger a self-amplifying inflammatory loop that exacerbates neuronal damage and amyloid deposition. Growing evidence suggests that several natural compounds can restore this balance. Compounds such as Ganoderic acid A (GAA) and Paeoniflorin (PF) suppress Th17 activation by inhibiting the JAK2/STAT3 signaling pathway and protect against neurotoxicity. Quercetin interferes with JAK/STAT signaling by blocking IL-12-induced tyrosine phosphorylation, thereby reducing downstream STAT3/STAT4 activation. Matrine downregulates the Th17-defining factor RORgt and upregulates the Treg-defining factor Foxp3. Luteolin reduces cortical A deposition and suppresses reactive astrogliosis. Epigallocatechin-3-gallate (EGCG) modulates amyloid metabolism by inhibiting BACE1 activity while simultaneously promoting Treg activity and suppressing Th17 differentiation. Targeting the Th17/Treg axis via immunometabolic modulation is a particularly promising avenue with high therapeutic potential.

A comprehensive review by Singh et al. (Contribution 5) identifies a significant intersection between long-term heavy alcohol consumption and the pathological processes that drive AD. Heavy alcohol consumption leads to microtubule instability, activating kinases such as GSK-3, which cause Tau proteins to detach and form neurofibrillary tangles (NFTs). It also disrupts the expression of the amyloid beta precursor (APP) and its processing enzyme BACE1, leading to increased Ab deposition. Alcohol activates microglial cells and increases the release of pro-inflammatory cytokines such as TNF-α and IL-1β. It also acts as a redox amplifier, increasing reactive oxygen species (ROS) production via cytochrome P450 2E1 (CYP2E1). Chronic alcohol consumption affects the structural integrity and functionality of different brain regions, including the hippocampus (memory consolidation), prefrontal cortex (executive function), amygdala (emotional regulation), and cerebellum (coordination). Chronic intake of alcohol inhibits the release of acetylcholine (Ach), a neurotransmitter essential for learning and memory. Given the deterministic impact of excessive alcohol intake on brain structure and function, the study advocates maintaining a healthy lifestyle and limiting alcohol intake as key measures to support brain health and mitigate the risk of neurodegenerative decline.

The authors conclude that chronic alcohol consumption acts as a catalyst for accelerated cerebral aging by triggering biological processes that intersect with the pathogenesis of AD.

A narrative review by Marongiu et al. (Contribution 6) explores the transformative potential of Artificial Intelligence (AI) to enhance diagnostic accuracy, prediction, and clinical management of AD using Positron Emission Tomography (PET). This review synthesizes recent scientific advances in the application of AI to three key PET modalities: 18-F-FDG PET, Amyloid PET, and Tau PET. The authors analyzed 455 research articles published between 2005 and early 2025, noting an exponential growth in research activity, particularly since 2017, driven by increased computing power, algorithmic development, and the availability of large-scale datasets. AI model frameworks can efficiently integrate multitracer PET data to improve sensitivity and specificity for early AD detection, predict conversion from Mild Cognitive Impairment (MCI) to AD, and track disease progression. Authors also pointed out that integrating PET neuroimaging with other data sources, such as structural MRI, CSF biomarkers, clinical information, and genetic profiles, will provide a more comprehensive outline of the brain’s pathological state.

A Machine Learning (ML) and Deep Learning (DL) approach possesses transformative potential to enhance the diagnostic and predictive utility of Positron Emission Tomography (PET) in the management of AD.

In their review, Hernandez Rodriguez and colleagues (Contribution 7) provide a critical assessment of how non-transgenic animal models can better represent sporadic Alzheimer’s disease (SAD). SAD accounts for 95% of all AD cases. The authors argue that despite decades of research and significant investment, effective treatments have not yet reached the market. Most of the candidate drugs fail in human clinical trials, despite showing success in transgenic animal models, because transgenic animal models are based on the less common familial form of the disease. Non-transgenic animal models of AD are positioned as a valuable and complementary strategy alongside transgenic models. Streptozotocin induces cerebral insulin resistance, glucose hypometabolism, and progressive cholinergic deficiency, leading to Aβ aggregates and Tau phosphorylation. Scopolamine administration causes memory impairment via cholinergic dysfunction and increased phosphorylated Tau levels by upregulating GSK3β. Senescence-accelerated mice (SAMP8) display various clinical features of age-related pathology, such as increased oxidative stress, and histopathological features, including Aβ plaques and neurofibrillary tangle formation, by 7–8 months of age. A mechanical stress-metal toxicity-high-fat diet model induced low-grade inflammation, insulin resistance, and Aβ deposition and Tau aggregation. Ultimately, they prioritized models that reflect real-world risk factors, which will be crucial for facilitating successful drug development and enhancing our understanding of SAD.

Wang et al. (Contribution 8) evaluate the therapeutic potential of photo-biomodulation (PBM) as a non-invasive alternative to traditional pharmacological treatment for AD. PBM utilizes low-intensity red or near-infrared light (600–1200 nm), which facilitates cellular repair. Mitochondrial cytochrome c oxidase (CCO) absorbs light at 600–900 nm, thereby increasing ATP production and cellular metabolism. PBM activates neuroprotective pathways, such as AKT, which inhibits the kinase GSK3B, implicated in cell death and Tau hyperphosphorylation, and increases brain-derived neurotrophic factor (BDNF) levels to promote neuronal health. Preclinical studies on AD mouse models (APP/PS1 and 5xFaD) demonstrated that PBM significantly reduces beta-amyloid plaques and Tau tangles. Notably, research suggests that PBM is particularly effective during sleep, possibly due to enhanced toxin clearance via the brain’s lymphatic system during deep sleep. A small-scale human pilot, double-blind study reported significant improvements in executive function, cognition, and neuroplasticity. Reviews emphasize that PBM is a promising, well-tolerated therapy that can mitigate several aspects of AD pathology, including inflammation and cognitive impairment. However, they also pointed out that large-scale, randomized, double-blind clinical trials are essential to establish definitive dosimetry guidelines and confirm its long-term safety and efficacy before it can be integrated into clinical practice.

A narrative review by Folia and Silva (Contribution 9) examines the efficacy of semantic memory interventions in the context of healthy aging and dementia. The authors explore how addressing semantic memory is crucial, as its efficiency directly affects other cognitive domains and everyday activities. The authors compared two primary methodologies used in cognitive programs: task-focused and person-centered approaches. Task-focused approaches are highly standardized, script-based cognitive intervention procedures designed to maintain or improve cognitive function, particularly in individuals with AD and MCI. These methodologies are characterized by adherence to controlled, predefined sequences of steps and by using predefined semantic materials, such as specific stories, word lists, or discussion topics, as the basis for training. On the other hand, person-centered approaches are cognitive intervention strategies that value the participant’s unique input rather than following a rigid, predefined script, prioritize expressive language, and encourage the individual to share their thoughts, memories, opinions, or feelings through written or oral communication. The author concluded that while both approaches are promising, there is a lack of direct comparison between them in existing literature. Future research should focus on validating individual semantic tasks and optimizing communication-based interventions.

A systematic review by Dognini et al. (Contribution 10) highlights a promising, non-invasive, and cost-effective tool for early and differential diagnosis of Mild Cognitive Impairment (MCI). The most consistent finding across the literature was that short-latency afferent inhibition (SAI), a marker of cholinergic dysfunction, was significantly reduced in individuals with amnestic MCI (aMCI) and MCI due to Alzheimer’s disease (MCI-AD). This makes SAI an early indicator for those at higher risk for AD. While SAI is more specific to AD, other TMS indices, such as short-interval intracortical inhibition (SICI) and intracortical facilitation (ICF), markers of dysfunction in GABAergic and glutamatergic pathways, were primarily altered in non-AD types of MCI. TMS achieves diagnostic accuracy of up to 91.3% for distinguishing MCI-AD from other types of MCI, comparable to amyloid PET scans and cerebrospinal fluid analysis. Including TMS data in the diagnostic workup significantly increased clinicians’ diagnostic confidence when identifying various MCI subtypes. In conclusion, TMS offers a safe, cost-effective, and accessible screening tool that can complement current biomarker workflows for MCI diagnosis. However, despite its potential, the authors emphasize that substantial work remains before it can be integrated into routine clinical practice.

Johnstone et al. (Contribution 11) found that white matter hyperintensities (WMHs) partially mediate the relationship between chronically elevated blood glucose levels (HbA1c) and cognitive impairment. This finding supports a potential microvascular pathway for diabetes-related cognitive impairment, suggesting that metabolic dysfunction, reflected by high HbA1c, leads to small-vessel damage in the brain and results in cognitive deficits. The researchers found a significant negative correlation between HbA1c levels and Montreal Cognitive Assessment (MoCA) scores, meaning higher average blood sugar was associated with poorer cognitive performance. Conversely, they found a positive correlation between HbA1c levels and WMH burden. Their mediation analysis revealed that WMHs partially mediate the relationship between HbA1c and cognitive function. Specifically, white matter damage accounts for approximately 15.6% of the total negative impact that HbA1c has on MoCA scores. The authors concluded that monitoring and controlling HbA1c levels could potentially decrease the risk of cognitive decline, possibly by limiting WMH accumulation.
